# Metabolomic and Lipidomic Profiling for Pre-Transplant Assessment of Delayed Graft Function Risk Using Chemical Biopsy with Microextraction Probes

**DOI:** 10.3390/ijms252413502

**Published:** 2024-12-17

**Authors:** Natalia Warmuzińska, Kamil Łuczykowski, Iga Stryjak, Emilia Wojtal, Aleksandra Woderska-Jasińska, Marek Masztalerz, Zbigniew Włodarczyk, Barbara Bojko

**Affiliations:** 1 Department of Pharmacodynamics and Molecular Pharmacology, Faculty of Pharmacy, Collegium Medicum in Bydgoszcz, Nicolaus Copernicus University in Torun, 85-089 Bydgoszcz, Poland; 2 Department of Transplantology and General Surgery, Faculty of Medicine, Collegium Medicum in Bydgoszcz, Antoni Jurasz University Hospital No. 1 in Bydgoszcz, Nicolaus Copernicus University in Torun, 85-094 Bydgoszcz, Poland

**Keywords:** solid-phase microextraction, LC-MS, kidney transplantation, metabolomics, lipidomics, graft quality assessment, DGF

## Abstract

Organ shortage remains a significant challenge in transplantology, prompting efforts to maximize the use of available organs and expand the donor pool, including through extended criteria donors (ECDs). However, ECD kidney recipients often face poorer outcomes, including a higher incidence of delayed graft function (DGF), which is linked to worse graft performance, reduced long-term survival, and an increased need for interventions like dialysis. This underscores the urgent need for strategies to improve early DGF risk assessment and optimize post-transplant management for high-risk patients. This study conducted multi-time point metabolomic and lipidomic analyses of donor kidney tissue and recipient plasma to identify compounds predicting DGF risk and assess the translational potential of solid-phase microextraction (SPME) for graft evaluation and early complication detection. The SPME-based chemical biopsy enabled a direct kidney analysis, while thin-film microextraction facilitated high-throughput plasma preparation. Following high-performance liquid chromatography coupled with a mass spectrometry analysis, the random forest algorithm was applied to identify compounds with predictive potential for assessing DGF risk before transplantation. Additionally, a comparison of metabolomic and lipidomic profiles of recipient plasma during the early post-operative days identified metabolites that distinguish between DGF and non-DGF patients. The selected compounds primarily included amino acids and their derivatives, nucleotides, organic acids, peptides, and lipids, particularly phospholipids and triacylglycerols. In conclusion, this study highlights the significant translational potential of chemical biopsies and plasma metabolite analyses for risk assessments and the non-invasive monitoring of DGF. The identified metabolites provide a foundation for developing a comprehensive DGF assessment and monitoring method, with potential integration into routine clinical practice.

## 1. Introduction

Kidney transplantation is a life-saving procedure for patients with end-stage renal disease, offering improved survival rates and quality of life compared to dialysis. However, the persistent shortage of available organs has resulted in a significant increase in the number of patients on kidney transplant waiting lists [1,2]. This growing imbalance between supply and demand has prompted clinicians to consider kidneys from extended criteria donors (ECDs) and donation after circulatory death (DCD) as alternatives. Unfortunately, recipients of ECD kidneys often experience poorer outcomes than those receiving organs from standard criteria donors, with a higher incidence of delayed graft function (DGF) and primary nonfunction [3]. DGF is a form of acute renal failure and one of the most frequent early complications in kidney transplant recipients. It is characterized by post-transplantation oliguria, increased allograft immunogenicity, and a higher risk of acute rejection episodes. DGF is associated with poor graft outcomes, decreased long-term survival, prolonged hospitalization, and the need for additional treatments such as dialysis. Both donor-related factors and prerenal, renal, or postrenal transplant factors in the recipient can contribute to the development of this condition [4,5]. The incidence of DGF is anticipated to rise in the coming years due to the increasing use of kidneys from ECDs and DCDs [4]. This trend is further influenced by the changing demographics of aging in human society, leading to a growing proportion of older donors and recipients, which may compound the risks associated with graft quality and post-transplant outcomes. Consequently, there is an urgent need for new strategies to enable early DGF risk assessment and optimize the management of high-risk recipients after transplantation [2,4]. Conventional methods for evaluating renal allografts, including donor medical history, visual inspection, and laboratory tests, often lack the specificity needed to predict the risk of DGF and make accurate diagnoses. While renal biopsy can provide valuable insights into pre-existing donor conditions and vascular changes, as well as help differentiate acute tubular necrosis (ATN)—the main cause of DGF in kidney transplant recipients—from other graft dysfunctions, it remains an invasive procedure [2,4].

In this study, metabolomic and lipidomic analyses were performed at multiple time points on donor kidney tissue and plasma samples from organ recipients to detect changes related to DGF. Metabolomics and lipidomics provide valuable insights into the organism’s immediate biochemical responses to various changes, helping to identify compounds associated with conditions such as ischemia, oxidative stress, inflammation, and other metabolic alterations that may contribute to graft dysfunction and complications in transplant recipients [6,7]. Solid-phase microextraction (SPME) chemical biopsy was employed for direct kidney analysis. The probe’s small diameter (∼200 μm) ensures minimal invasiveness and allows for multiple samplings from the same organ without causing tissue damage. Additionally, thin-film microextraction (TFME) was used as a high-throughput plasma sample preparation method. SPME is a versatile and highly sensitive sample preparation technique that integrates sampling, extraction, and analyte concentration into a single step [7,8]. The combination of kidney graft and recipient plasma analysis aimed to identify compounds with predictive potential for assessing DGF risk and to evaluate the translational applicability of SPME for graft quality assessments and the early detection of complications.

## 2. Results

### 2.1. Demographic Characteristics and Clinical Data

The donors’ and recipients’ demographic characteristics and clinical parameters are presented in Table 1 and Table 2, respectively. Donors were grouped based on the donor type (extended criteria donor (ECD) or standard criteria donor (SCD)), while recipients were classified based on the occurrence of DGF. The last available data were collected for donors prior to organ procurement and for recipients from the final tests conducted before hospital discharge following transplantation.

A statistically significant age difference was observed among donors, as age is one of the primary criteria for categorizing a donor as an ECD. Additionally, SCD donors had significantly lower platelet (PLT) counts compared to ECD donors.

Among recipients, significantly higher creatinine and blood urea nitrogen (BUN) levels and significantly lower glomerular filtration rate (GFR) values were observed in those who developed DGF, indicating that these patients still exhibited poorer kidney function just before their discharge from the hospital.

Furthermore, an additional analysis was conducted to determine whether differences existed in the laboratory test results of donors whose kidneys were associated with DGF in recipients (Table S1); however, no statistically significant differences were observed.

### 2.2. Metabolomic and Lipidomic Analyses

Metabolomic and lipidomic analyses were conducted on the donor’s kidney tissue and plasma samples from organ recipients to identify compounds with predictive potential in assessing DGF risk and to evaluate the translational applicability of SPME for graft quality assessments and the early detection of complications. A PCA was employed to confirm the quality of the instrumental analysis for all combinations of chromatographic separation and ionization mode. As shown in Supplementary Figure S1, the pooled quality control (QC) samples formed a tight cluster, confirming the high quality of the analytical performance. For clarity, the results section is divided into two parts: the first part focuses on the assessment of graft quality using a chemical biopsy, while the second part presents the analysis of plasma samples collected from recipients on days 1 and 5 post-operation.

### 2.3. Assessment of Graft Quality Using Chemical Biopsy

To identify compounds with potential predictive value for the early detection of DGF risk, kidney cortex samples were collected using a minimally invasive chemical biopsy at three time points: after organ procurement, prior to transplantation, and during reperfusion. Due to the imbalanced distribution of DGF complication occurrences in the study group, a light oversampling process was applied to the minority cases (DGF) to improve the stability and quality of the modelling results. This process was carried out separately for each of the three time points to maintain the temporal characteristics of the sample and minimize the risk of overfitting the model to the artificially augmented data. The Random Over-Sampling Examples (ROSE) algorithm was used, allowing for the generation of additional observations in the minority group by creating synthetic examples based on the classification variable. For each time point, the sample size of the DGF group was increased to 15, achieving a DGF to non-DGF ratio of 15:22.

An initial selection of metabolites was conducted using the random forest algorithm with 500 decision trees, separately for each time point. Compounds with the highest mean decrease accuracy (MDA) and mean decrease Gini (MDG) values were identified. A list of the 10 metabolites with the highest MDA and MDG values for each analytical block is presented in Table S2. Subsequently, compounds with both high MDA and MDG values were chosen from the preselected set for further model development and included in Table 3. Among the selected compounds were mainly amino acids and their derivatives, organic acids, and lipids, particularly phospholipids and triacylglycerols (TGs).

**Figure 1:**
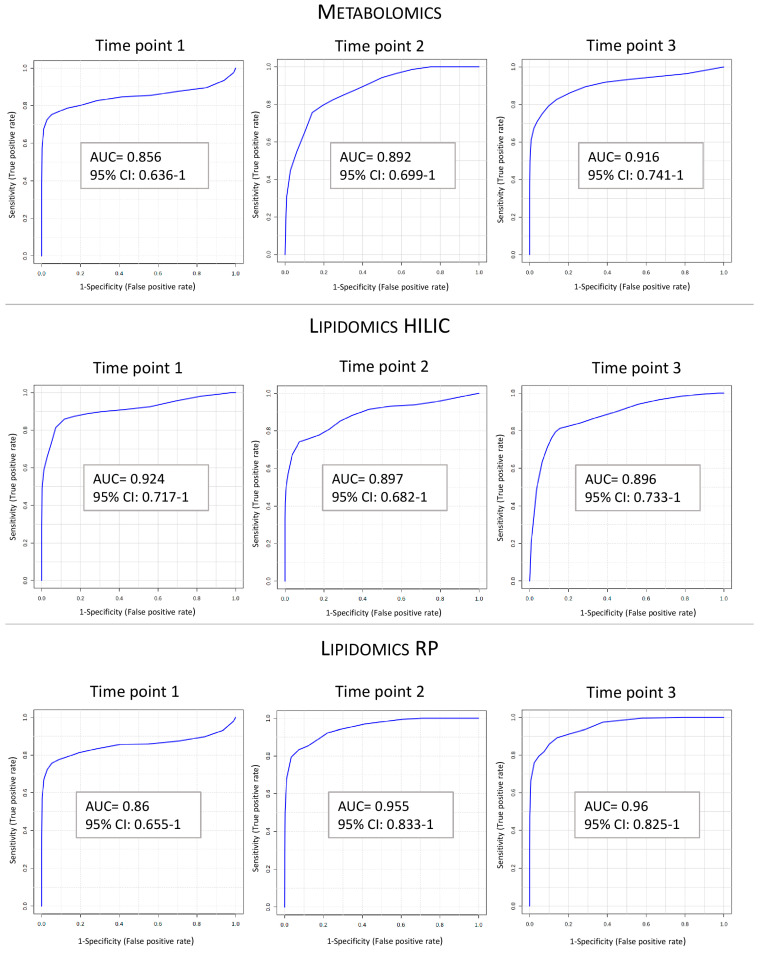
Receiver operating characteristic (ROC) curves of random forest models for selected compound sets distinguishing between DGF and non-DGF groups. AUC: area under curve.

Random forest models based on the selected metabolites were evaluated using 10-fold cross-validation to identify the optimal *mtry* value. After determining the optimal *mtry*, the model was retrained with this value, using 10-fold cross-validation, to assess its predictive performance. The model evaluation metrics are presented in Table 4. To visualize the performance of the obtained models, receiver operating characteristic (ROC) curves were generated using the Biomarker Analysis module in MetaboAnalyst 6.0 (Figure 1). The nine predictive models demonstrated a consistent performance across several key metrics. The accuracy ranged from 0.79 to 0.91, indicating a high rate of correct predictions across the models. Cohen’s kappa values varied between 0.55 and 0.82, reflecting a strong level of agreement beyond chance. Sensitivity values ranged from 0.73 to 0.93, highlighting the models’ ability to reliably detect true positive cases. Specificity values were between 0.77 and 1, indicating a strong performance in correctly classifying true negatives. The precision ranged from 0.73 to 1, and F1-scores varied from 0.73 to 0.88, suggesting a balanced model performance with a good trade-off between precision and recall. Additionally, the area under the ROC curve (AUC) for all models exceeded 0.85, further confirming their strong predictive capabilities. Based on the consistent performance of the models across various metrics, it can be inferred that the selected compounds may have predictive potential for assessing the risk of DGF development.

**Figure 2:**
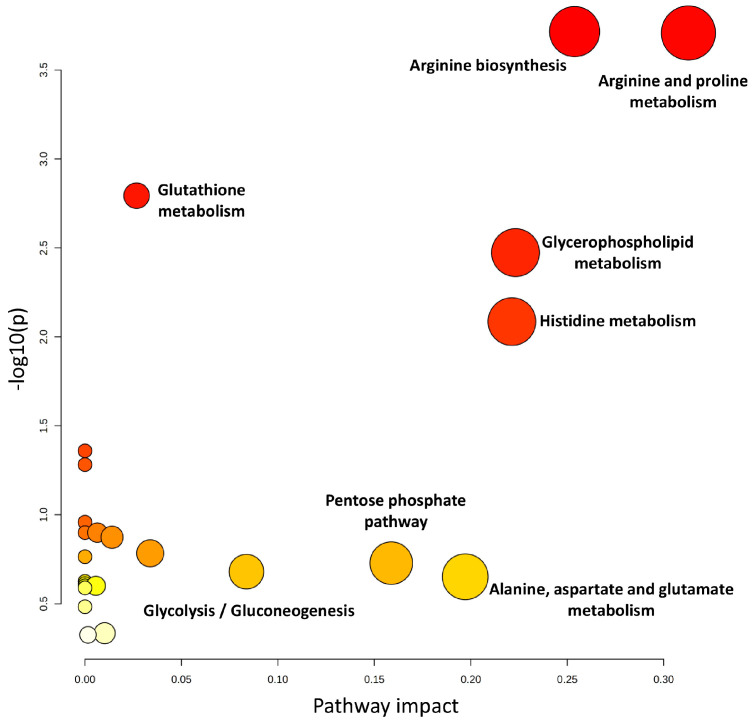
Pathway analysis of metabolites selected for random forest models from metabolomic and lipidomic analyses.

Furthermore, a pathway analysis was conducted to explore the biological context of the results. This analysis was performed using the list of selected metabolites, with the results presented in Figure 2. The complete list of identified pathways is provided in Table S3.

### 2.4. Analysis of Plasma Samples Collected from Recipients on Days 1 and 5 Post-Operation

**Figure 3:**
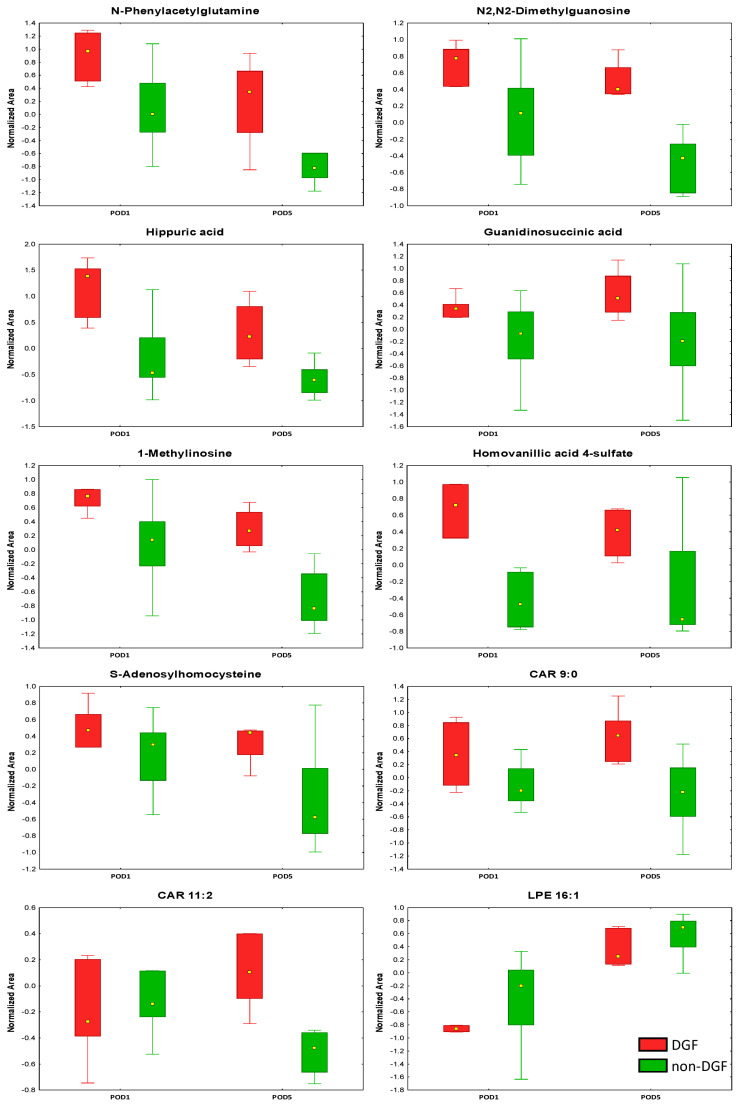
Selected metabolites differentiating the DGF and non-DGF groups on post-operative days 1 and 5. The rectangle’s height represents the normalized peak areas in the interquartile range (Q1 and Q3). The upper whisker denotes the largest data point (excluding any outliers), while the lower whisker denotes the lowest data point (excluding any outliers). The median normalized peak area of each group is indicated with a yellow square.

A plasma analysis was performed to identify metabolites that differentiate samples from non-DGF and DGF patients. Differential metabolites were selected through a Volcano plot, combining the fold change (FC > two) and a nonparametric Mann–Whitney U test (*p* < 0.05) to ensure selection based on both biological and statistical significance. Analyses were conducted separately for samples collected on post-operative days 1 and 5 (POD1 and POD5, respectively), and the results are summarized in Table 5 and Figure S2. Boxplots of selected statistically significant metabolites are shown in Figure 3. Of the 49 identified compounds, 42 showed elevated levels in the DGF patient group. A greater number of differentiating compounds were observed on POD5. Additionally, metabolites that distinguished the study groups on both sampling days exhibited significantly higher levels on POD5. The identified metabolites are classified into various groups based on their chemical structure, including amino acids, peptides, phenolic acids, lipids, nucleotides, and their derivatives. These molecules play a crucial role in various biological functions, ranging from energy metabolism and fatty acid transport to hormonal regulation and the elimination of toxic metabolic byproducts. A pathway analysis was performed using the list of significantly different compounds identified in the metabolomic and lipidomic analyses. The results are presented in Figure S3 and Table S4.

## 3. Discussion

DGF is one of the most common early complications in kidney transplant recipients, associated with poor graft outcomes, an increased risk of acute rejection, prolonged hospitalization, and the need for additional medical interventions, such as dialysis [4]. Recent research highlights that, beyond its mere occurrence, the duration of DGF may exert the most profound impact on graft outcomes [9,10]. Consequently, there is a pressing need to develop novel methods for early DGF risk assessment and the effective management of high-risk recipients post-transplantation. In recent years, significant efforts have been made to identify potential biomarkers for the early and minimally invasive detection of DGF, as well as to evaluate donor-, recipient-, and surgery-related risk factors [4]. However, conventional diagnostic approaches, such as serum creatinine or GFR measurements, lack specificity, while organ biopsy remains highly invasive [2,4]. To maintain a minimally invasive approach, most studies focus on identifying biomarkers in urine, plasma, or perfusion fluid [4]. Direct tissue analysis is rarely pursued due to the invasive nature of traditional sample preparation techniques, which require the excision of tissue fragments.

The method proposed in this study—chemical biopsy—represents a minimally invasive technique that avoids tissue damage and allows for repeated direct tissue analyses. This approach, combined with a random forest algorithm, allowed for the selection of compounds with potential predictive value for DGF occurrence, which can be evaluated prior to organ transplantation. Furthermore, metabolomic and lipidomic analyses of recipient plasma enabled the identification of compounds differentiating DGF patients during the initial post-operative days.

Among the metabolites forming the basis of the predictive models developed in this study are those previously described in the context of kidney function and peri-transplantation graft performance assessment. In recent years, many studies have attempted to define the role of specific amino acids and their derivatives in kidney function and damage. This work demonstrated that, among this group of compounds, arginine, ornithine, glutamic acid, and histidine may exhibit potential predictive roles in the occurrence of DGF. Ansermet et al. revealed that a possible explanation for the worsening of mitochondrial function in the ischemic kidney may be provided by the metabolic role of arginase 2 (ARG2)-generated ornithine in the proximal tubule [11]. ARG2 in the kidney co-localizes with ornithine aminotransferase, an enzyme catalyzing the conversion of ornithine to glutamate, which can be deaminated to α-ketoglutarate. The latter may improve the energy deficit in ischemia–reperfusion injury (IRI) by serving as a substrate in the anaerobic adenosine triphosphate (ATP) synthesis pathway in the proximal tubule. This highlights the protective role of ornithine in renal pathology and its potential therapeutic implications in renal transplantation [11,12]. Similarly, arginine and ornithine have been implicated as key amino acids in understanding the mechanisms involved in IRI following liver transplantation [13]. Histidine has also been shown to be an amino acid whose levels in body fluids are correlated with kidney status and was more sensitive to any short-term changes in renal activity than creatinine [14]. A reduced urinary histidine level indicated impaired kidney function, while a low serum histidine level was characteristic for chronic kidney disease (CKD) patients [15,16].

The next metabolites identified in this study were 4-Hydroxynonenal (4-HNE) and 4-oxononenal, two of the most thoroughly studied lipid peroxidation products [17]. The presence of these compounds may indicate oxidative stress as an important factor in kidney damage and dysfunction. Increased lipid peroxidation, measured by an elevation in 4-HNE, was observed in the kidneys for ischemia- or drug-induced acute kidney injury (AKI) [18,19]. In another study, Wang et al. showed that the disruption of mitochondrial energy production by the inhibition of the mitochondrial respiratory chain resulted in increased oxidative stress, leading to glomerular filtration barrier damage [20].

In recent years, adenosine has also been widely studied in the context of renal function and kidney damage. Its role as a signaling molecule in various physiological processes, including renal blood flow regulation and cellular protection during IRI, makes it a critical factor in kidney transplantation outcomes. Crikis et al. showed that CD39 expression on either endothelial cells or circulating immune cells enhances adenosine generation and follows adenosine A_2A_ receptor signaling, contributing to reduced apoptosis under warm ischemia injury and extended cold preservation [21]. However, the role of the ectoenzyme CD73 remains unclear, as research findings suggest both adenosine and adenosine monophosphate as mediators of protection against IRI [22,23]. In a recent paper, Wang et al. reviewed the adenosinergic metabolism pathway as an emerging target for enhancing outcomes in solid organ transplantation [24].

The selected lipids with potential predictive value were primarily TGs, phosphatidylcholine (PC), phosphatidylethanolamine (PE), and sphingomyelin (SM). Lipids belonging to these groups have been previously reported in the literature in relation to graft quality assessment and kidney diseases [25,26,27]. Changes in TG levels have been associated with the progression of CKD and impaired mitochondrial β-oxidation [27], which is the preferred source of ATP in the kidneys [28]. Furthermore, in our previous studies evaluating different donor types in an animal model, changes in TG levels were also observed [25]. The accumulation of neutral lipids in cells is typically associated with lipotoxicity; however, the renal toxicity of TGs depends on their acyl chain length and the number of double bonds [29,30]. TGs may act as a reservoir for excess free fatty acids that have been removed from cells, thereby preventing fatty-acid-induced lipotoxicity [31].

PCs, PEs, and SMs are essential for cellular structure and function. PCs and PEs are the most abundant phospholipids found in mammalian cells and their subcellular organelles, playing a critical role in membrane integrity and signaling pathways [32]. Changes in the levels of PCs, PEs, and SMs have previously been reported in animal models of IRI [33,34], chronic kidney disease [27,35], acute graft rejection [36], and diabetic nephropathy [37], as well as in studies comparing different kidney preservation strategies [26]. Changes in the levels of PCs and/or PEs may alter the overall PC/PE ratio, which can, in turn, influence processes in various organelles. For instance, a lower PC/PE ratio has been shown to impair liver regeneration, disrupt energy metabolism, increase cell membrane permeability, and induce endoplasmic reticulum stress [32,37].

TFME has already been used for the analysis of body fluids to identify low-molecular-weight metabolites that may serve as potential biomarkers of diseases or pathophysiological conditions [38,39]. In this study, a number of compounds were identified in the plasma that differentiate non-DGF patients from those with DGF. The metabolomic profiles of non-DGF and DGF patients differed already on the first day after transplantation. Furthermore, on POD5, more significant differences in profiles were observed, through the detection of additional metabolites and an increase in the fold changes of those previously identified.

Many of the metabolites identified in this study are considered uremic toxins—organic or inorganic substances that accumulate in the body fluids of individuals with impaired renal function, including acute or chronic renal disease. Sato et al., conducting a metabolomic analysis of plasma from hemodialysis patients, demonstrated that toxins such as 1-methylinosine, N2,N2-dimethylguanosine, phenylacetylglutamine, and hippuric acid can be used as alternatives to urea and creatinine in assessing organ function [40]. In another study, Grams et al. also identified N2,N2-dimethylguanosine, hippuric acid, and homovanillic acid 4-sulfate as molecules associated with CKD [41]. Hippuric acid is one of the most well-characterized uremic toxins, shown to be elevated in hemodialysis patients with chronic renal failure compared to both healthy controls and hospitalized patients without renal disease [42]. Sun et al. revealed that hippuric acid promotes the progression of renal fibrosis by disrupting redox homeostasis, which is maintained by the nuclear factor erythroid 2-related factor 2 (NRF2) antioxidant network [43].

Another uremic toxin, guanidinosuccinic acid (GSA), is primarily derived from the metabolism of arginine and plays a role in the urea cycle. In patients with kidney dysfunction, GSA levels can rise significantly due to impaired renal clearance. Zhang et al. demonstrated that GSA levels were markedly elevated in rats exposed to chronic low-dose cadmium, suggesting that GSA could serve as a biomarker for kidney damage [44]. Furthermore, Gu et al. reported a significant increase in GSA concentrations in a rat model of renal failure, indicating that this metabolite may serve as an early biomarker for nephrotoxicity, potentially preceding traditional markers such as creatinine and uric acid [45].

In this work, one of the differentiating metabolites was S-Adenosylhomocysteine (SAH), a direct precursor of all homocysteine produced in the body, formed through the demethylation of S-adenosyl-L-methionine. The correlation between elevated SAH levels in serum or urine and renal dysfunction or insufficiency in patients with end-stage renal disease and CKD has been frequently reported in previous studies [46,47,48,49]. SAH is a potent inhibitor of the trans-methylation enzymes, and the accumulation of SAH in plasma might affect the trans-methylation pathway in CKD patients [47,50]. Given that SAH is a potential marker of cardiovascular and renal dysfunction, Klepacki et al. developed and validated an LC-MS method for determining SAH in plasma as an early marker of acute rejection and nephrotoxicity in kidney transplant recipients. Their study showed that samples collected during or prior to clinical events affecting the kidney graft, such as biopsy-proven acute allo-immune reactions and immunosuppressive drug nephrotoxicity, showed significantly higher concentrations of SAH compared to samples from transplant patients during periods of stable kidney graft function [51].

Among the compounds showing higher levels in the DGF recipient group, acylcarnitines (CARs) were also identified. Changes in acylcarnitine levels have been previously associated with various kidney conditions, including AKI, the progression of CKD, and diabetic nephropathy, as documented in several studies [15,26,27,52,53]. CARs play a critical role in transporting long-chain fatty acids into mitochondria, facilitating the β-oxidation process [52,54]. Elevated plasma levels of acylcarnitines indicate mitochondrial damage, while under non-pathological conditions, their concentrations remain low [54,55]. Moreover, elevated CARs levels have been previously observed in patients with reduced GFR, highlighting their association with impaired kidney function [15,56].

**Figure 4:**
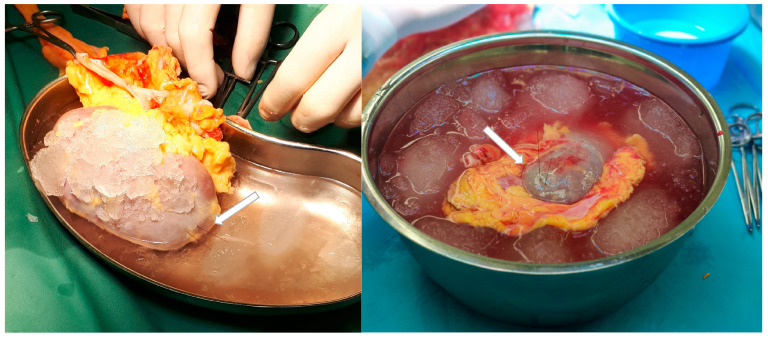
Chemical biopsy sampling during simultaneous surgical preparation of the graft.

The obtained results demonstrate significant translational potential, which is worth emphasizing. Due to the small diameter of the probe and the lack of a need for physical tissue collection, chemical biopsy enables multiple direct analyses of kidney tissue. This capability, which is challenging to achieve with other methods, is coupled with the preservation of the procedure’s minimally invasive nature. Direct tissue analysis provides organ-specific insights, which are particularly significant when assessing the quality of kidneys intended for transplantation. Notably, kidneys from the same donor may vary in their characteristics and exhibit different levels of risk for the development of DGF or other complications in the recipient. Biomarker evaluation in recipients’ urine or blood presents a promising alternative to current laboratory tests for diagnosing and monitoring post-transplant complications, offering potentially greater levels of sensitivity and specificity. However, for the early identification of complication risks and better management of high-risk patients, it is crucial to develop methods capable of assessing these risks at the stage of graft quality evaluation. The use of a needle biopsy for organ quality assessments varies across medical facilities and countries. For example, while up to 85% of high-risk kidneys undergo biopsies in the United States, pre-transplant biopsies are rarely performed in European centers [2,57]. Furthermore, existing studies assessing the predictive value of renal biopsies concerning graft outcomes have yielded predominantly inconclusive results [58,59,60,61]. Another challenge is the low reproducibility of biopsy evaluations between on-call and renal pathologists, as highlighted in previous studies [62]. Additionally, the invasive nature of the procedure often limits biopsies to a single sampling plan. Although the translational potential of chemical biopsy has been previously highlighted [25,26], to the best of our knowledge, this study is the first to apply a chemical biopsy for kidney graft quality assessments in a clinical setting. Undoubtedly, the results obtained in this study are highly promising and suggest the potential for incorporating chemical biopsy into routine diagnostic practices. From a practical standpoint, sampling performed immediately after organ procurement and after preservation but before transplantation may have the greatest utility. This approach would allow for the assessment of donor-related risk factors and, in cases of doubt or prolonged cold ischemia time, the opportunity to re-evaluate the risk of complications. Moreover, the 10-min extraction used in this study did not prolong the ischemic time of the organ, as the small size of the probes allowed for the simultaneous surgical preparation of the graft (Figure 4). Furthermore, we assume that by applying a targeted analysis on a validated biomarker panel, it will be possible in the future to further shorten the extraction time. Combined with the monitoring of plasma metabolites, this could lead to the development of a comprehensive method for assessing risk and monitoring complications in kidney transplant recipients.

However, despite these promising results, this study has several limitations that should be considered. The primary limitation is the relatively small sample size and the imbalanced groups. Given the complexity of DGF’s underlying causes, further studies with larger sample sizes are needed to validate the results obtained in this study. Moreover, studies conducted on a larger patient cohort should consider the impact of the organ preservation method and other clinical parameters on the assessment of DGF risk, which was not achievable in this study due to the small sample size. Additionally, the lack of metabolomic and lipidomic profiling of recipients’ blood before transplantation is a limitation, along with the relatively short follow-up period of 5 days post-transplant. Future research should aim to verify the predictive models developed in this study in the context of long-term outcomes.

## 4. Materials and Methods

### 4.1. Chemicals

Reagents, including water (LC/MS Optima grade), ammonium acetate, formic acid, and acetic acid, were sourced from Merck (Poznan, Poland), while acetonitrile, methanol, and isopropanol (all LC/MS Optima grade) were supplied by Alchem (Torun, Poland). Pierce LTQ Velos ESI Positive External Calibrant Solution and Negative Ion Calibration Solution were purchased from Anchem (Anchem, Warsaw, Poland). Prototypes of biocompatible SPME mixed-mode (MM) probes (C18 and benzenesulfonic acid) were kindly provided by Supelco (Bellefonte, PA, USA).

### 4.2. Patients

**Figure 5:**
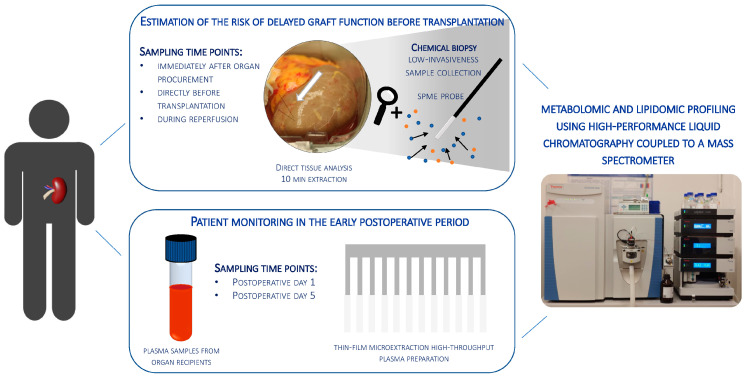
Study design. The study involved samples collected from kidneys recovered from deceased donors and plasma samples from organ recipients.

This study received prior approval from the Bioethics Committee in Bydgoszcz (KB 636/2017), and informed consent was obtained from all subjects. The study involved samples collected from kidneys recovered from deceased donors (n = 24) and plasma samples from organ recipients. The study protocol is illustrated in Figure 5. A total of 32 kidneys were sampled at multiple time points, while plasma samples were obtained from 20 recipients. The condition for including an organ in the study cohort was its qualification for transplantation at Antoni Jurasz University Hospital No. 1 in Bydgoszcz. Decisions were made by the transplant team based on the donor’s medical history, laboratory parameters, and, in doubtful cases, kidney biopsy results. The study group included SCD and ECD. The kidneys were preserved using static cold storage or hypothermic machine perfusion. The patient cohort was divided into two groups based on the occurrence of DGF in the organ recipient. The DGF patient group consisted of patients in whom ATN was confirmed by biopsy (n = 7) and those diagnosed with DGF based on clinical data (n = 3). These two types of DGF patients were combined to allow for a dichotomous definition of DGF. Clinical parameters were collected from donors before organ procurement and from recipients during final tests conducted prior to hospital discharge following transplantation. Donors’ laboratory tests were performed at the centers where the kidneys were procured, while recipients’ tests were conducted at Antoni Jurasz University Hospital No. 1 in Bydgoszcz. Hematological tests were carried out using the Sysmex XN system, and biochemical parameters were measured with dedicated assays on the Alinity c Abbott system.

### 4.3. Sample Collection

Kidney tissue sampling was performed using an SPME probe with a 4 mm coating of MM sorbent as the extraction phase. Before use, the probes were conditioned in a 50:50 methanol:water solution at 850 rpm for 30 min to pre-activate the sorbent, ensuring optimal extraction efficiency, followed by steam sterilization in accordance with standard procedures for medical tools and equipment sterilization. Metabolite extraction, referred to as chemical biopsy, was carried out by carefully inserting the probe into the renal cortex for 10 min at three specific time points: immediately after organ procurement (time point 1), directly before transplantation (time point 2), and during reperfusion (time point 3). Following extraction, the probes were rinsed with distilled water to remove residual blood, placed into empty glass vials, transported on ice, and stored at −80 °C until instrumental analysis. Blood samples from recipients were collected in sodium citrate tubes on POD1 and POD5. After centrifugation, the resulting plasma was aliquoted and stored in Eppendorf tubes at −80 °C until instrumental analysis.

### 4.4. Sample Preparation

Two distinct desorption solutions were employed to desorb analytes from SPME probes and tailored to the specific analytical requirements of the study. For metabolomic analysis, a solution composed of acetonitrile and water (50:50, *v*/*v*) was utilized. Conversely, for lipidomic analysis, a mixture of isopropanol and methanol (50:50, *v*/*v*) was applied. In both analytical approaches, desorption was conducted in a total volume of 200 µL of the respective desorption solution. The desorption process was carried out for 2 h under agitation at 850 rpm using a BenchMixer™ MultiTube Vortexer (Benchmark Scientific, Edison, NJ, USA). To control for potential contamination, extraction blanks were prepared. These blanks consisted of fibers processed according to the same protocol as the sampling fibers (including preconditioning and desorption), but they were not exposed to kidney samples.

Plasma sample preparation was conducted using SPME, with each step of the process performed on a high-throughput 96-manual TFME system (Professional Analytical System (PAS) Technology, Magdala, Germany). This system enabled the simultaneous analysis of all samples [38]. For metabolomic analyses, extractions were performed using stainless blades coated with hydrophilic–lipophilic balanced (HLB) sorbent, specifically N-vinylpyrrolidone-divinylbenzene copolymer (Alchem, Toruń, Poland). In contrast, for lipidomic studies, blades coated with C18 sorbent (Alchem, Toruń, Poland) were utilized. In both cases, the coating length was 5 mm. The coating preparation procedure followed the spray method described by Mirnaghi et al. [63]. Steel blades were procured from PAS Technology (Magdala, Germany), while polypropylene Nunc 96-well DeepWell plates were obtained from Merck (Poznań, Poland).

Before initiating the extraction, the SPME blades were conditioned for 30 min in 0.5 mL of a methanol:water (50:50, *v*/*v*) solution in 96-well plates, with agitation at 850 rpm. Following conditioning, a 10-s wash step was performed. Extractions were performed from 0.5 mL of plasma spiked with acetylcarnitine-d_4_ (Merck, Poland) for metabolomics and SPLASH (Merck, Poland) for lipidomics, respectively, with agitation at 850 rpm for 1 h. After extraction, the blades were placed in 0.5 mL of nanopure water for 10 s. Following the wash step, desorption was conducted in 0.5 mL organic solutions identical to those used for the desorption of SPME probes with agitation (850 rpm) for 2 h.

### 4.5. LC-MS Analysis

Samples were analyzed using an LC-HRMS procedure involving the coupling of an ultra-high performance liquid chromatograph (Dionex UHPLC system) and a Q-Exactive Focus Orbitrap mass spectrometer (Thermo Fisher Scientific, Bremen, Germany). Data acquisition was performed using dedicated Thermo Scientific software, namely, Xcalibur 4.2 and Free Style 1.4 (Thermo Fisher Scientific, San Jose, CA, USA). The instrument was externally calibrated every 72 h, achieving a mass accuracy of <2 ppm. To ensure consistent performance, within-sequence samples were randomized, and pooled QC samples (comprising 10 μL of each sample) were analyzed every 8–10 injections.

### 4.6. Metabolomics Analysis

Chromatographic separation was carried out in reversed-phase (RP) mode using a pentafluorophenyl column (Supelco Discovery HS F5, 2.1 mm × 100 mm, 3 μm). Samples collected via chemical biopsy were analyzed in positive ionization mode, while plasma samples were analyzed in both positive and negative ionization modes. Detailed parameters of the method, including the mobile phases and gradient employed, have been previously described in [25]. The mass spectrometer parameters in positive ionization mode were as follows: a sheath gas flow rate of 40 a.u.; an aux gas flow rate of 15 a.u.; a spray voltage of 3.5 kV; a capillary temp of 300 °C; an aux gas heater temp of 300 °C; an S-lens radio frequency level of 55%; an S-lens voltage of 25 V; and a skimmer voltage of 15 V. In negative ionization mode, the mass spectrometer parameters were as follows: a sheath gas flow rate of 48 a.u.; an aux gas flow rate of 11 a.u.; a spray voltage of 2.5 kV; a capillary temp of 256 °C; an aux gas heater temp of 413 °C; an S-lens radio frequency level of 55%; an S-lens voltage of −25 V; and a skimmer voltage of −15 V. The scan range was set to 80–1000 m/z with a resolution of 70,000 full width at half maximum (FWHM). Acquisition was performed using an automatic gain control (AGC) target of 1E6, with the C-trap injection time set to auto. The putative identification of compounds was confirmed based on full MS/dd-MS2 mode. The fragmentation parameters were set as follows: mass resolution—35,000 FWHM; AGC target—2E4; minimum AGC—8E3; intensity threshold—auto; maximum IT—auto; isolation window—3.0 m/z; stepped collision energy—10 V, 20 V, and 40 V; loop count—2; and dynamic exclusion—auto. Fragmentation spectra were verified using online databases including LIPID MAPS, Human Metabolome Database, METLIN, and mzCloud.

### 4.7. Lipidomics Analysis

Chromatographic separation was carried out on a hydrophilic stationary-phase (HILIC) column (SeQuant ZIC-cHILIC, 3 μm, 100 × 2.1 mm) and in RP using a C18 column (Waters, XSelect CSH C18, 3.5 µm, 2.1 × 75 mm). The analyses were performed in positive electrospray ionization mode. Detailed information on the chromatographic separation and mass spectrometer parameters has been previously described in [26]. The putative identification of compounds was confirmed in full MS/dd-MS2 mode using the following fragmentation parameters: mass resolution—35,000 FWHM; AGC target—2E4; minimum AGC—8E3; intensity threshold—auto; maximum IT—auto; isolation window—3.0 m/z; stepped collision energy—20 V, 30 V, and 50 V; loop count—2; and dynamic exclusion—auto.

### 4.8. Data Processing and Statistical Analysis

The raw metabolomics data were processed using Compound Discoverer 3.1 (Thermo Fisher Scientific, San Jose, CA, USA) software to identify metabolites present in the samples. Detected metabolites with a signal-to-noise ratio > 3 and a peak intensity > 100,000 were subjected to further analysis. The intensity tolerance was set at 30%, and the RT tolerance was set at 0.2 min. The QC-based area was used for correction (min 80% coverage and max 30% RSD in QC). After peak alignment, gap filling was performed to fill in the missing values via a very small peak at the level of spectrum noise for the compound.

Raw lipidomics data were processed separately using LipidSearch 4.1.30 (Thermo Fisher Scientific, San Jose, CA, United States) software with the following parameters: peak intensity >100,000; a precursor tolerance of 5 ppm; a product tolerance of 10 ppm; an m-score threshold of 2; a Quan m/z tolerance of ±5 ppm; a Quan RT (retention time) range of 0.5 min; and the use of a main isomer filter. H+, NH4+, and Na+ adducts were considered in positive ionization mode. After lipid identification, the alignment process was conducted using LipidSearch with the following parameters: an m-score threshold of 10; a retention time tolerance of 0.25 min; a QC-to-extraction-blank ratio of >5; and a max 30% RSD in the QC. The search mode function of the software sought matches of parent peaks (full scan MS) and product peaks (fragments, MS/MS) with the lipid database entries. The software’s search mode matched parent peaks (full scan MS) and product peaks (fragments, MS/MS) with the lipid database entries. Each feature was assigned a grade (A–D) based on its quality, with grade filtering applied to eliminate false positives. During alignment, only lipids identified with grades A to C were retained.

The results from each LC-HRMS block constituted separate datasets and were analyzed independently. Peak areas for the identified compounds were processed using MetaboAnalyst 6.0, Statistica 13.3 PL (StatSoft, Inc., Tulsa, OK, USA) and RStudio (version 2024.9.0+375, Posit Team, 2024) with R version 4.4.1 (R Core Team, 2024). All missing values were replaced with small values assumed to represent the detection limit. Data obtained from chemical biopsy were normalized using the probabilistic quotient normalization (PQN) method, followed by log transformation and Pareto scaling. Plasma data were normalized using an internal standard, followed by log transformation and Pareto scaling. Principal component analysis (PCA) was conducted to visually confirm the quality of the analytical performance.

To evaluate the significance of changes, the Student’s *t*-test was used for parametric data, while the Mann–Whitney U test was applied to nonparametric data. Homogeneity was assessed using Levene’s test, and normality was checked with the Shapiro–Wilk test. Additionally, Fisher’s exact test was used to examine associations between categorical variables. A *p*-value of <0.05 was considered statistically significant.

For metabolomic and lipidomic data obtained from chemical biopsy sampling, the following R packages were used for data preprocessing and model development: ROSE, to address class imbalance; RandomForest, to construct the random forest model; and caret, for model training and cross-validation. To visualize the performance of the resulting models, ROC curves were generated using the Biomarker Analysis module in MetaboAnalyst 6.0. The same platform was also used for pathway analysis, employing the Homo sapiens Kyoto Encyclopedia of Genes and Genomes (KEGG) metabolic pathway database.

## 5. Conclusions

In conclusion, the findings of this study highlight the significant translational potential of chemical biopsy and plasma metabolite analysis for assessing the risk and the non-invasive monitoring of DGF. The compounds identified in this research provide a basis for future studies aimed at validating their predictive value in a larger cohort. Upon the completion of studies involving a broader patient population, it is anticipated that a comprehensive method for assessing and monitoring DGF will be developed, with the potential for integration into routine clinical practice.

## Figures and Tables

**Table 1:** Deceased donor characteristics, stratified by donor type.

Characteristic	Total (n = 24)	SCD (n = 19)	ECD (n = 5)	*p*-Value
Age, year	44.5 [41.5–47.5]	44 [41–46]	60 [60–66]	0.02980
Sex				
Male	17 (71%)	12 (63%)	5 (100%)	>0.05
Hypertension	3 (13%)	3 (16%)	0	>0.05
Cause of death				
Trauma	9 (38%)	7 (37%)	2 (40%)	>0.05
Vascular	12 (50%)	9 (47%)	3 (60%)	>0.05
Others	3 (13%)	3 (16%)	0	
Creatinine, mg/dL	1.27 [0.98–2.13]	1.40 [1.05–2.39]	1.20 [0.60–1.24]	>0.05
Urea, mg/dL	45.64 [37.20–62.00]	44.00 [34.10–51.80]	62.00 [48.00–100.00]	>0.05
Procalcitonin, ng/mL	1.37 [0.57–8.18]	1.11 [0.57–7.96]	2.05 [1.37–13.35]	>0.05
CRP, mg/L	246.60 (135.13)	226.29 (126.44)	323.81 (153.78)	>0.05
WBC, 10^3^/µL	12.98 [11.05–17.40]	13.00 [11.78–16.70]	10.40 [10.20–18.70]	>0.05
HGB, g/dL	11.11 (2.64)	11.54 (2.64)	9.46 (2.11)	>0.05
PLT, 10^3^/µL	142.88 (57.46)	130.37 (46.54)	190.40 (75.31)	0.03436
AST, IU/L	52.00 [29.00–95.00]	37.00 [26.00–98.00]	74.50 [52.00–88.00]	>0.05
ALT, IU/L	50.00 [23.00–90.00]	46.00 [23.00–90.00]	55.50 [45.5–81.50]	>0.05
K, mmol/L	4.40 [3.70–4.68]	4.40 [3.80–4.66]	4.07 [3.00–4.90]	>0.05
Na, mmol/L	155.91 (11.04)	157.26 (11.23)	150.78 (9.59)	>0.05
Cl, mmol/L	117.17 (10.91)	119.60 (10.35)	109.38 (9.72)	>0.05

SCD: standard criteria donor, ECD: extended criteria donor.

**Table 2:** Kidney transplant recipient characteristics, stratified by DGF occurrence.

Characteristic	Total (n = 32)	Non DGF (n = 22)	DGF (n = 10)	*p*-Value
Age, year	57.5 [43.5–64]	57.50 [48–64]	55 [41–63]	>0.05
Sex				
Male	25 (78%)	17 (77%)	8 (80%)	>0.05
Hypertension	27 (84%)	18 (82%)	9 (90%)	>0.05
Donor criteria				>0.05
SCD	26 (81%)	20 (91%)	6 (60%)	>0.05
ECD	6 (19%)	2 (9%)	4 (40%)	>0.05
Preservation method				
HMP	11 (34%)	8 (36%)	3 (30%)	>0.05
SCS	21 (66%)	14 (64%)	7 (70%)	>0.05
Recipients requiring dialysis	13 (40%)	5 (23%)	8 (80%)	0.00506
Bacteria in preservative fluid	12 (37.5%)	9 (41%)	3 (30%)	>0.05
Ischemia time	17 h 26 min (7 h 16 min)	16 h 03 min (7 h 06 min)	20 h 47 min (6 h 54 min)	>0.05
Creatinine, mg/dL	1.59 (0.58)	1.34 (0.41)	2.14 (0.54)	0.00006
GFR, mL/min/1.73 m^2^	50.49 (15.96)	57.77 (13.13)	34.46 (7.72)	0.00001
BUN, mg/dL	31.80 [21.80–38.40]	24.70 [21.40–33.80]	45.00 [35.60–56.50]	0.00098
CRP, mg/L	3.62 [2.48–6.06]	4.98 [3.02–6.23]	2.43 [0.68–3.48]	>0.05
WBC, 10^3^/µL	8.32 (3.05)	8.62 (3.38)	7.66 (2.15)	>0.05
HGB, g/dL	10.37 (0.91)	10.27 (0.83)	10.60 (1.07)	>0.05
PLT, 10^3^/µL	229.97 (77.25)	228.41 (76.74)	233.40 (82.41)	>0.05
AST, IU/L	14.00 [11.00–17.00]	14.50 [11.00–19.50]	13.00 [10.50–15.00]	>0.05
ALT, IU/L	15.50 [13.00–29.50]	16.50 [13.00–32.00]	14.50 [11.00–27.00]	>0.05
K, mmol/L	4.42 (0.46)	4.41 (0.52)	4.44 (0.31)	>0.05
Na, mmol/L	141.87 (2.95)	141.78 (3.21)	142.06 (2.41)	>0.05
Cl, mmol/L	110.10 [107.00–112.10]	110.60 [107.30–112.20]	109.90 [107.00–111.00]	>0.05
Ca, mmol/L	2.23 (0.22)	2.26 (0.21)	2.18 (0.25)	>0.05

SCD: standard criteria donor, ECD: extended criteria donor, HMP: hypothermic machine perfusion, SCS: static cold storage.

**Table 3:** Compounds selected for further model development based on MDA and MDG values.

Name	MW	RT	Mean Decrease Accuracy	Mean Decrease Gini	*p*-Value	AUC	Fold Change
**Metabolomics**							
Time point 1							
Pyroglutamic acid	129.04	1.75	8.752	1.551	0.002	0.803	4.478
4-oxo-2-nonenal	154.10	10.09	6.484	0.917	0.053	0.692	0.471
N-Butyryl-L-homoserine lactone	171.09	1.74	5.937	0.859	0.013	0.746	0.419
Ornithine	132.09	1.88	5.731	0.889	0.003	0.787	2.735
Glutaric acid	132.04	1.32	4.705	0.474	0.026	0.721	0.375
Tetramethylpyrazine	136.10	8.95	3.819	0.601	0.039	0.704	1.613
Heptylic acid	130.10	1.69	3.305	0.506	>0.05	0.651	0.983
Time point 2							
Phosphoethanolamine	141.01	11.70	5.746	0.613	0.029	0.715	0.385
Glutamic acid	147.05	1.53	5.577	0.683	0.011	0.748	0.219
Glyceraldehyde 3-phosphate	170.00	2.11	5.243	0.913	0.005	0.779	2.387
Histidine	155.07	1.90	5.065	0.644	>0.05	0.624	1.677
1-Nitronaphthalene-5,6-oxide	189.04	24.76	4.992	0.581	0.019	0.73	1.843
Adenosine	267.10	7.28	4.805	0.643	>0.05	0.645	0.776
Carnitine	161.11	3.35	4.369	0.852	0.006	0.767	0.201
Time point 3							
Phosphoethanolamine	141.01	11.70	9.547	1.966	0.0003	0.852	0.029
3-Indolebutyric acid	203.10	13.52	6.979	1.343	0.0009	0.824	3.763
NA-Val 18:0	383.34	22.19	6.040	0.896	0.026	0.718	1.596
4-Hydroxynonenal	156.12	11.86	5.431	0.606	>0.05	0.552	1.533
Creatine	131.07	1.98	4.971	0.735	0.011	0.748	0.016
Adenosine	267.10	7.28	4.912	0.806	>0.05	0.561	15.12
Arginine	174.11	2.012	4.456	0.636	>0.05	0.679	0.34
**Lipidomics HILIC**							
Time point 1							
SM 42:1;O2	814.69	7.78	6.180	1.053	0.003	0.79	2.326
PC 38:5	807.58	6.48	5.532	0.638	0.028	0.717	0.091
PC 34:1	759.58	6.53	5.511	0.813	0.001	0.784	0.07
SM 40:2;O2	784.65	7.94	5.046	0.536	>0.05	0.66	0.616
PC 38:6	805.56	6.49	4.820	0.459	>0.05	0.663	4.379
LPC 18:0	523.36	8.84	4.474	0.467	>0.05	0.657	0.621
SM 34:1;O2	702.57	8.16	4.317	0.483	>0.05	0.625	0.954
PE 38:5	765.53	7.42	4.289	0.549	>0.05	0.673	1.471
Time point 2							
PE 38:5	765.53	7.56	7.547	0.981	>0.05	0.578	1.371
PC 34:1	759.58	6.62	6.284	0.636	>0.05	0.681	2.574
PC P-34:2	741.57	6.58	4.900	0.514	0.02	0.727	4.991
PE 38:6	763.52	7.45	4.734	0.673	>0.05	0.654	1.912
SM 40:1;O2	786.66	7.91	4.687	0.567	>0.05	0.678	0.434
PE 36:3	741.53	7.45	4.670	0.495	>0.05	0.596	1.823
PC 38:5	807.58	6.48	4.280	0.468	>0.05	0.668	0.28
LPE 18:0	481.32	10.34	4.040	0.519	>0.05	0.681	0.644
Time point 3							
PE 38:6	763.52	7.45	6.126	0.854	>0.05	0.591	1.313
PE 36:3	741.53	7.45	5.718	0.716	>0.05	0.591	1.231
PC 36:4	781.56	6.52	5.468	0.756	>0.05	0.652	1.029
PE 36:2	743.55	7.56	5.324	0.660	0.024	0.721	2.392
PE 36:4	739.52	7.46	4.985	0.655	>0.05	0.657	0.982
PC 36:3	783.58	6.49	4.715	0.589	>0.05	0.506	0.887
PC 34:2	757.56	6.69	4.289	0.493	>0.05	0.648	0.29
**Lipidomics RP**							
Time point 1							
TG 56:0	918.86	14.63	4.684	0.500	0.003	0.79	2.087
TG 48:5	796.66	12.52	4.027	0.456	0.014	0.743	1.401
TG P-52:1	846.80	14.11	3.750	0.295	>0.05	0.502	1.486
Cer 34:0;O2	539.53	10.70	3.701	0.249	>0.05	0.575	0.537
TG 50:1	832.75	13.77	3.282	0.247	>0.05	0.571	0.704
Cer 32:0;O2	511.50	9.99	3.247	0.293	>0.05	0.581	0.653
Time point 2							
TG 56:0	918.86	14.63	5.891	0.650	0.0007	0.833	3.7405
DG 36:4	616.51	10.03	5.395	0.654	>0.05	0.606	1.6009
DG 36:3	618.52	10.46	4.257	0.376	>0.05	0.627	1.201
TG O-52:1	846.80	14.35	4.099	0.274	>0.05	0.603	3.0758
TG 44:3	744.63	12.81	3.955	0.252	>0.05	0.675	1.993
TG 42:0	722.64	12.81	3.807	0.269	0.0268	0.718	2.675
TG 50:0	834.77	13.99	3.676	0.266	>0.05	0.518	2.397
TG 54:2	886.80	14.08	3.258	0.287	>0.05	0.666	3.003
Time point 3							
DG 34:1	594.52	10.73	5.148	0.642	>0.05	0.645	0.5487
TG 56:2	914.83	14.30	4.383	0.378	>0.05	0.645	1.334
TG 54:1	888.81	14.24	4.256	0.404	>0.05	0.524	0.2857
TG 52:0	862.80	14.23	4.221	0.388	>0.05	0.603	0.0913
TG 55:2	900.81	14.13	4.041	0.326	0.042	0.7	0.4624
TG 54:6	878.74	13.94	3.921	0.261	>0.05	0.615	0.7927
TG 50:2	830.74	13.57	3.615	0.236	>0.05	0.533	2.1772
TG 56:3	912.81	14.08	3.608	0.238	>0.05	0.612	0.8343

MW: molecular weight, RT: retention time (min), AUC: area under curve, SM: sphingomyelin, PC: phosphatidylcholine, LPC: lysophosphocholine, PE: phosphatidylethanolamine, LPE: lysophosphatidylethanolamine, TG: triacylglycerol, Cer: ceramide, DG: diglyceride.

**Table 4:** Random forest models’ evaluation metrics.

	Metabolomics		
	Time Point 1	Time Point 2	Time Point 3
**Accuracy**	0.83	0.86	0.78
**Kappa**	0.66	0.72	0.55
**Sensitivity**	0.80	0.87	0.73
**Specificity**	0.86	0.86	0.82
**Precision**	0.80	0.81	0.73
**F1-Score**	0.80	0.84	0.73
	**Lipidomics HILIC**		
	**Time Point 1**	**Time Point 2**	**Time Point 3**
**Accuracy**	0.89	0.89	0.89
**Kappa**	0.77	0.78	0.78
**Sensitivity**	0.87	0.93	0.87
**Specificity**	0.90	0.86	0.91
**Precision**	0.87	0.82	0.87
**F1-Score**	0.87	0.88	0.87
	**Lipidomics RP**		
	**Time Point 1**	**Time Point 2**	**Time Point 3**
**Accuracy**	0.92	0.84	0.86
**Kappa**	0.82	0.68	0.72
**Sensitivity**	0.80	0.93	0.87
**Specificity**	1.00	0.77	0.86
**Precision**	1.00	0.74	0.81
**F1-Score**	0.89	0.82	0.84

**Table 5:** The list of statistically significant compounds in comparisons of DGF and non-DGF patients on POD1 and POD5.

Name	MW	RT	DGF vs. Non-DGF Patients			
			POD1		POD5	
			FC	*p*-Value	FC	*p*-Value
**Metabolomics—RP positive ionization mode**						
Indole-3-acryloylglycine	244.08	11.70	8.20	0.001	19.32	0.040
4-Hydroxyprolylisoleucine	244.14	9.09	2.92	0.001	n.a	n.a
N-Phenylacetylglutamic acid	265.09	9.90	6.40	0.003	12.94	0.010
3-hydroxydecanoyl carnitine	331.24	18.08	3.22	0.003	2.13	0.026
N-Nonanoylglycine	215.15	9.29	2.98	0.003	4.92	0.018
1-Methylinosine	282.10	6.94	2.97	0.003	2.66	0.018
1-Methylhypoxanthine	150.05	6.94	2.62	0.003	2.95	0.018
Hippuric acid	179.06	8.96	3.95	0.005	9.22	0.006
5-Hydroxyindole	133.05	9.72	3.84	0.005	n.a	n.a
Phenylacetamide	135.07	8.84	3.73	0.005	4.63	0.022
N-Phenylacetylglutamine	264.11	8.85	3.05	0.007	5.72	0.022
Tyramine	137.08	2.13	2.02	0.007	n.a	n.a
Indoleacetyl glutamine	303.12	9.74	2.94	0.013	11.95	0.003
Cinnamoylglycine	205.07	11.48	2.79	0.018	14.38	0.040
N-Phenylacetylaspartic acid	251.08	9.77	2.70	0.018	2.61	0.040
L-Urobilin	590.31	15.71	3.82	0.024	n.a	n.a
alpha-Aminoadipic acid	161.07	1.58	n.a	n.a	2.77	0.010
3-Methylxanthine	166.05	6.66	n.a	n.a	8.09	0.006
1-Methyluric acid	182.04	6.68	n.a	n.a	4.96	0.010
2-Keto-glutaramic acid	145.04	1.56	n.a	n.a	2.95	0.010
N2,N2-Dimethylguanosine	311.12	7.31	n.a	n.a	3.12	0.010
Leu-Leu	244.18	14.54	n.a	n.a	0.28	0.018
Pro-Leu	228.15	8.54	n.a	n.a	3.06	0.018
Ala-Leu	202.13	7.32	n.a	n.a	3.33	0.018
9,11alpha-epoxypregn-4-ene-3,20-dione	328.20	11.31	n.a	n.a	2.51	0.018
Cys-Trp	307.10	11.42	n.a	n.a	2.43	0.018
Formiminoglutamic acid	174.06	2.019	n.a	n.a	2.09	0.026
Trp-Phe	351.16	19.58	n.a	n.a	0.43	0.040
Guanidinosuccinic acid	175.06	1.65	n.a	n.a	2.21	0.040
**Metabolomics—RP negative ionization mode**						
Indole-3-acryloylglycine	244.08	11.70	5.21	0.002	17.05	0.006
N-Phenylacetylglutamic acid	265.09	9.90	4.54	0.007	13.14	0.040
4-Hydroxyprolylleucine	244.14	9.18	2.82	0.009	n.a	n.a
Homovanillic acid 4-sulfate	262.01	7.85	4.09	0.046	n.a	n.a
Gluconic acid	196.06	7.76	2.78	0.046	n.a	n.a
Indoleacetyl glutamine	303.12	9.74	2.77	0.046	10.16	0.003
3-indole carboxylic acid glucuronide	337.08	10.00	n.a	n.a	4.95	0.003
Indoxyl glucuronide	309.08	8.97	n.a	n.a	6.90	0.010
Trp-Phe	351.16	19.58	n.a	n.a	0.43	0.010
11beta-Hydroxyandrosterone-3-glucuronide	482.25	12.12	n.a	n.a	5.97	0.018
Tetrahydroaldosterone-3-glucuronide	540.26	11.42	n.a	n.a	3.00	0.018
Asp-Phe	280.11	9.88	n.a	n.a	7.12	0.026
Deoxycholic Acid	392.29	16.47	n.a	n.a	0.16	0.026
3,17-Androstanediol glucuronide	468.27	14.35	n.a	n.a	3.90	0.040
LPE 18:0	481.32	18.60	n.a	n.a	0.39	0.040
S-Adenosylhomocysteine	384.12	7.52	n.a	n.a	2.22	0.040
**Lipidomics—RP positive ionization mode**						
CAR 9:0	301.23	1.53	2.20	0.019	3.05	0.001
**Lipidomics—HILIC positive ionization mode**						
CAR 17:1	411.33	7.45	0.38	0.019	n.a	n.a
LPE 16:1	451.27	10.50	0.38	0.035	n.a	n.a
CAR 9:0	301.23	8.07	n.a	n.a	3.11	0.001
CAR 11:2	325.23	8.22	n.a	n.a	4.45	0.004
CAR 16:0	399.33	7.50	n.a	n.a	2.54	0.037
SPB 18:0;O2	301.3	10.23	n.a	n.a	2.65	0.048
CAR 14:0	371.30	7.65	n.a	n.a	2.13	0.048

MW: molecular weight, RT: retention time (min), FC: fold change, Leu: leucine, Pro: proline, Ala: alanine, Cys: cysteine, Trp: tryptophan, Phe: phenylalanine, Asp: aspartic acid, LPE: lysophosphatidylethanolamine, CAR: acylcarnitine, SPB: sphinganine, n.a.: not applicable.

## Data Availability

The datasets generated during and/or analyzed during the current study are available from the corresponding author on reasonable request.

## Supplementary Materials

The following supporting information can be downloaded at: https://www.mdpi.com/article/10.3390/ijms252413502/s1.

## Abbreviations

4-HNE 4-Hydroxynonenal; AGC automatic gain control; AKI acute kidney injury; Ala alanine; ARG2 arginase 2; Asp aspartic acid; ATN acute tubular necrosis; ATP adenosine triphosphate; AUC area under curve; BUN blood urea nitrogen; CAR acylcarnitine; Cer ceramide; CKD chronic kidney disease; Cys cysteine; DCD donation after circulatory death; DG diglyceride; DGF delayed graft function; ECD extended criteria donor; FC fold change; FWHM full width at half maximum; GFR glomerular filtration rate; GSA guanidinosuccinic acid; HILIC hydrophilic stationary phase; HLB hydrophilic–lipophilic balanced; HMP hypothermic machine perfusion; IRI ischemia-reperfusion injury; Leu leucine; LPC lysophosphocholine; LPE lysophosphatidylethanolamine; MDA mean decrease accuracy; MDG mean decrease Gini; MM mixed mode; NRF2 nuclear factor erythroid 2-related factor 2; PAS Professional Analytical System; PC phosphatidylcholine; PCA principal component analysis; PE phosphatidylethanolamine; Phe phenylalanine; PLT platelet; POD post-operative day; PQN probabilistic quotient normalization; Pro proline; QC quality control; ROC Receiver operating characteristic; ROSE Random Over-Sampling Examples; RP reversed-phase; SAH S-Adenosylhomocysteine; SCS static cold storage; SM sphingomyelin; SPB sphinganine; SPME solid-phase microextraction; TFME thin-film microextraction; TG triacylglycerol; Trp tryptophan.
