# Supplementary material for: Metabolomic and Lipidomic Profiling for Pre-Transplant Assessment of Delayed Graft Function Risk Using Chemical Biopsy with Microextraction Probes
Source: Int J Mol Sci. 2024 Dec 17;25(24):13502. doi: 10.3390/ijms252413502 (PMC11728147; doi:10.3390/ijms252413502)
Supplement: Supplementary file 1 [file ijms-25-13502-s001.zip › ijms-3368401-supplementary.pdf]

Supplementary Information

Metabolomic and lipidomic profiling for pre-transplant risk assessment of delayed graft function using chemical biopsy with microextraction probes

Natalia Warmuzińska[1], Kamil Łuczykowski[1], Iga Stryjak[1], Emilia Wojtal[2], Aleksandra Woderska-Jasińska[2], Marek Masztalerz[2], Zbigniew Włodarczyk[2], Barbara Bojko[1]

[1]Department of Pharmacodynamics and Molecular Pharmacology, Faculty of Pharmacy, Collegium Medicum in Bydgoszcz, Nicolaus Copernicus University in Torun, Bydgoszcz, Poland

[2]Department of Transplantology and General Surgery, Collegium Medicum in Bydgoszcz, Antoni Jurasz University Hospital No. 1 in Bydgoszcz, Nicolaus Copernicus University in Torun, Bydgoszcz, Poland

Table S1. Donors laboratory test results whose kidneys were associated with delayed graft function (DGF) in recipients.

Characteristic	Total (n=32)	non DGF (n=22)	DGF (n=10)	p-value
Ischemia time	17h26min (7h09min)	16h03min (7h06min)	20h27min (6h35min)	>0.05
Creatinine, mg/dL	1.27 [0.98-2.13]	1.35 [1.05-2.13]	1.15 [0.90-1.50]	>0.05
Urea, mg/dL	45.00 [37.20-62.00]	47.00 [37.20-90.00]	39.30 [24.70-48.00]	>0.05
CRP, mg/L	256.09 (127.38)	242.85 (134.01)	285.21 (112.29)	>0.05
Procalcitonin, ng/mL	1.37 [0.57-9.12]	5.35 [0.57-9.12]	1.07 [0.44-2.05]	>0.05
WBC, 10^3/uL	12.98 [11.75-18.40]	12.96 [11.70-16.70]	13.52 [11.78-20.10]	>0.05
HGB, g/dL	10.50 [8.80-13.75]	9.90 [8.50-14.00]	11.40 [10.20-12.60]	>0.05
PLT, 10^3/uL	145.50 [92.00-169.50]	146.00 [92.00-171.00]	139.00 [127.00-168.00]	>0.05
AST, IU/L	68.00 [31.00-98.00]	54.00 [29.00-95.00]	68.00 [52.00-132.00]	>0.05
ALT, IU/L	51.00 [26.00-103.00]	62.00 [26.00-109.00]	46.00 [45.00-60.00]	>0.05
K, mmol/L	4.21 (0.74)	4.22 (0.63)	4.20 (0.99)	>0.05
Na, mmol/L	156.59 (11.82)	154.24 (9.89)	161.77 (14.47)	>0.05
Cl, mmol/L	117.62 (11.63)	115.62 (10.06)	121.43 (13.93)	>0.05

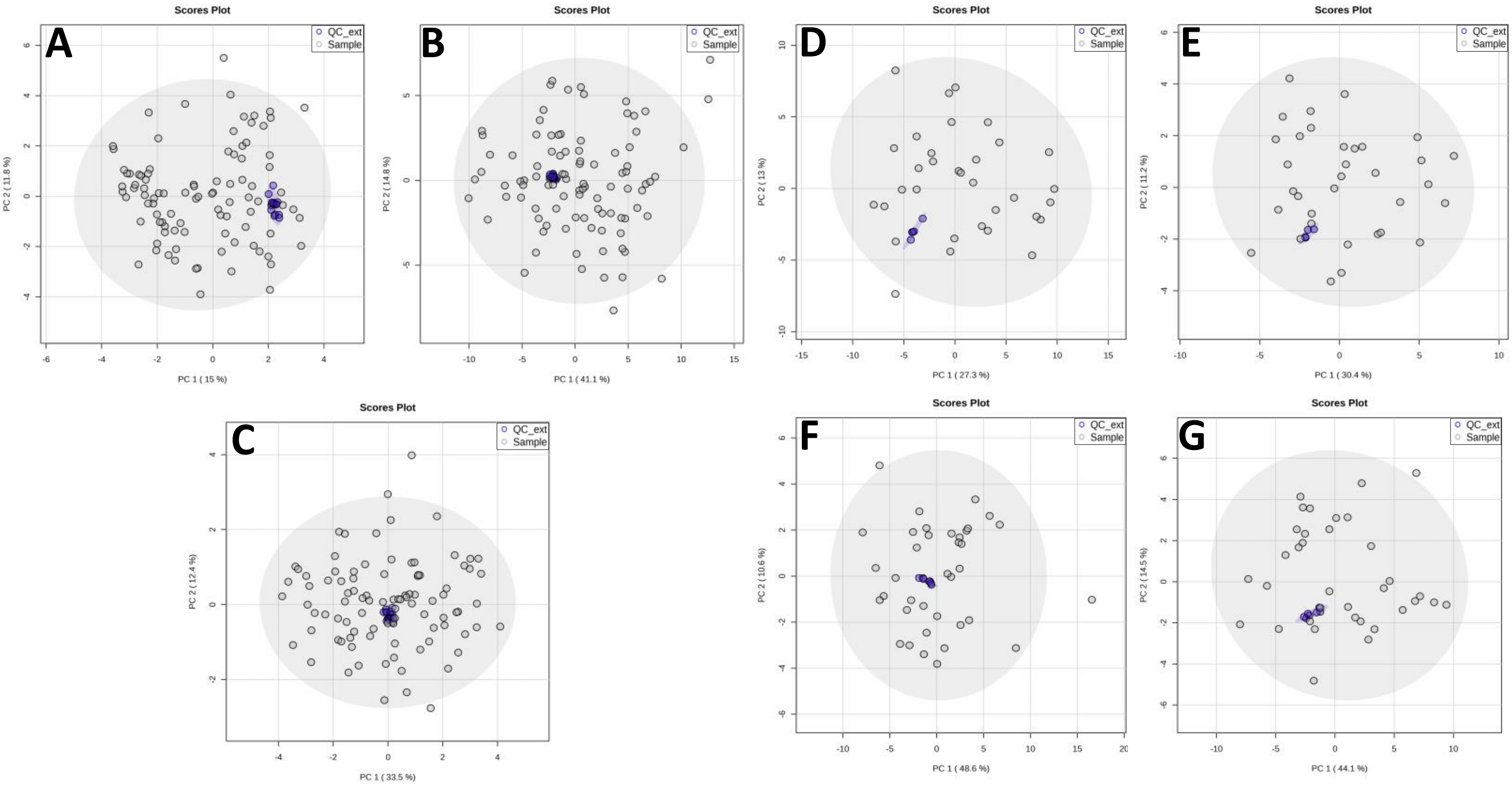

Figure S1. Principal component analysis (PCA) plots of all analyzed samples and extraction quality control (QC) samples for chemical biopsy analysis: (A) metabolomics, (B) Lipidomics RP, (C) Lipidomics HILIC, and plasma samples analysis: (D) Metabolomics – RP Positive ionization mode, (E) Metabolomics – RP Negative ionization mode, (F) Lipidomics - RP Positive ionization mode, (G) Lipidomics - HILIC Positive ionization mode.

Table S2. Metabolites with the highest MDA and MDG values for each analytical block.

Name	MW	RT	Mean Decrease Accuracy	Mean Decrease Gini
**Metabolomics**				
Time point 1				
Pyroglutamic acid	129.04	1.75	8.752	1.551
4-oxo-2-nonenal	154.10	10.09	6.484	0.917
N-Butyryl-L-homoserine lactone	171.09	1.74	5.937	0.859
Ornithine	132.09	1.88	5.731	0.889
Glutaric acid	132.04	1.32	4.705	0.474
Tetramethylpyrazine	136.10	8.95	3.819	0.601
Heptylic acid	130.10	1.69	3.305	0.506
Glyceraldehyde 3-phosphate	169.99	2.11	3.870	0.433
Carnitine	161.11	3.35	3.570	0.226
Dopamine	153.08	9.93	3.371	0.452
4-Nitroaniline	138.04	6.84	2.658	0.602
Leucine	131.09	9.14	1.558	0.533
Nicotine glucuronide	338.15	16.46	0.056	0.456
Time point 2				
Phosphoethanolamine	141.01	11.70	5.746	0.613
Glutamic acid	147.05	1.53	5.577	0.683
Glyceraldehyde 3-phosphate	169.99	2.11	5.243	0.913
Histidine	155.07	1.90	5.065	0.644
1-Nitronaphthalene-5,6-oxide	189.04	24.76	4.992	0.581
Adenosine	267.10	7.28	4.805	0.643
Carnitine	161.11	3.35	4.369	0.852
Homocycloleucine	143.09	1.75	5.215	0.553
3,5-Dihydroxypentanoic acid	134.06	1.73	4.545	0.296
7-Methyl-2,6,9,12-tetraoxahexadecane	248.20	14.09	4.523	0.358
NA-Val 18:0	383.34	22.19	4.090	0.607
5-Aminopentanoic acid	117.08	1.70	3.851	0.601
Tetramethylpyrazine	136.10	8.95	4.251	0.564
Time point 3				
Phosphoethanolamine	141.01	11.70	9.547	1.966
3-Indolebutyric acid	203.10	13.52	6.979	1.343
NA-Val 18:0	383.34	22.19	6.040	0.896
4-Hydroxynonenal	156.12	11.86	5.431	0.606
Creatine	131.07	1.98	4.971	0.735
Adenosine	267.10	7.28	4.912	0.806
Arginine	174.11	2.01	4.456	0.636
Dopamine	153.08	9.93	3.553	0.383
Taurine	125.01	1.26	3.092	0.261
3,5-Dihydroxypentanoic acid	134.06	1.73	3.006	0.251
Glyceraldehyde 3-phosphate	169.99	2.11	1.335	0.510
Heptylic acid	130.10	1.69	2.699	0.438
Pyroglutamic acid	129.04	1.75	2.747	0.389

Lipidomics HILIC				
Time point 1				
SM 42:1;O2	814.69	7.78	6.180	1.053
PC 38:5	807.58	6.48	5.532	0.638
PC 34:1	759.58	6.53	5.511	0.813
SM 40:2;O2	784.65	7.94	5.046	0.536
PC 38:6	805.56	6.49	4.820	0.459
LPC 18:0	523.36	8.84	4.474	0.467
SM 34:1;O2	702.57	8.16	4.317	0.483
PE 38:5	765.53	7.42	4.289	0.549
PE O-38:5	751.55	7.26	4.591	0.408
PE 34:1	717.53	7.46	4.248	0.399
PC O-34:2	743.58	6.59	2.375	0.547
PC 38:4	809.59	6.46	3.967	0.422
Time point 2				
PE 38:5	765.53	7.56	7.547	0.981
PC 34:1	759.58	6.62	6.284	0.636
PC P-34:2	741.57	6.58	4.900	0.514
PE 38:6	763.52	7.45	4.734	0.673
SM 40:1;O2	786.66	7.91	4.687	0.567
PE 36:3	741.53	7.45	4.670	0.495
PC 38:5	807.58	6.48	4.280	0.468
LPE 18:0	481.32	10.34	4.040	0.519
LPC P-16:0	479.34	8.58	4.035	0.363
PC 36:3	783.58	6.49	3.274	0.554
Time point 3				
PE 38:6	763.52	7.45	6.126	0.854
PE 36:3	741.53	7.45	5.718	0.716
PC 36:4	781.56	6.52	5.468	0.756
PE 36:2	743.55	7.56	5.324	0.660
PE 36:4	739.52	7.46	4.985	0.655
PC 36:3	783.58	6.49	4.715	0.589
PC 34:2	757.56	6.69	4.289	0.493
SM 40:2;O2	784.65	7.94	4.819	0.356
SM 36:1;O2	730.6	8.03	4.441	0.350
SM 40:1;O2	786.66	7.91	4.368	0.400
PE 40:7	789.53	7.42	3.929	0.841
PE P-36:1	729.57	7.38	4.188	0.528
PC 33:4	739.52	7.47	3.712	0.520
**Lipidomics RP**				
Time point 1				
TG 56:0	918.86	14.63	4.684	0.500
TG 48:5	796.66	12.52	4.027	0.456
TG P-52:1	846.80	14.11	3.750	0.295
Cer 34:0;O2	539.53	10.70	3.701	0.249
TG 50:1	832.75	13.77	3.282	0.247
Cer 32:0;O2	511.50	9.99	3.247	0.293

DG 36:3	618.52	10.46	3.361	0.186
TG 54:1	888.81	14.27	3.225	0.178
TG 53:3	870.77	13.75	3.085	0.157
TG 50:0	834.77	13.99	3.052	0.146
DG 36:2	620.54	10.88	3.029	0.375
TG 34:0	610.52	11.14	2.175	0.275
TG 46:5	768.63	12.63	2.497	0.244
DG 35:1	608.54	11.02	2.953	0.235
Time point 2				
TG 56:0	918.86	14.63	5.891	0.650
DG 36:4	616.51	10.03	5.395	0.654
DG 36:3	618.52	10.46	4.257	0.376
TG O-52:1	846.80	14.35	4.099	0.274
TG 44:3	744.63	12.81	3.955	0.252
TG 42:0	722.64	12.81	3.807	0.269
TG 50:0	834.77	13.99	3.676	0.266
TG 54:2	886.80	14.08	3.258	0.287
TG 54:1	888.81	14.27	3.348	0.138
TG 50:4	826.71	13.13	3.237	0.144
DG 36:2	620.54	10.88	2.556	0.287
DG 32:2	564.48	9.55	2.943	0.275
Time point 3				
DG 34:1	594.52	10.73	5.148	0.642
TG 56:2	914.83	14.30	4.383	0.378
TG 54:1	888.81	14.24	4.256	0.404
TG 52:0	862.80	14.23	4.221	0.388
TG 55:2	900.81	14.13	4.041	0.326
TG 54:6	878.74	13.94	3.921	0.261
TG 50:2	830.74	13.57	3.615	0.236
TG 56:3	912.81	14.08	3.608	0.238
TG 56:6	906.77	13.71	4.061	0.229
DG 55:2	886.84	13.61	3.700	0.191
TG 50:0	834.77	13.99	3.004	0.289
TG 55:4	896.78	14.16	3.379	0.288

Table S3. Results of pathway analysis of metabolites selected for Random Forest models from metabolomic and lipidomic analyses

Pathway name	Match status	p	FDR
Arginine biosynthesis	3/14	0.000192	0.007836
Arginine and proline metabolism	4/36	0.000196	0.007836
Glutathione metabolism	3/28	0.001608	0.042886
Glycerophospholipid metabolism	3/36	0.00336	0.067201
Histidine metabolism	2/16	0.008204	0.13126
Linoleic acid metabolism	1/5	0.043716	0.58288
Nitrogen metabolism	1/6	0.052243	0.59707
alpha-Linolenic acid metabolism	1/13	0.10999	0.93626
Butanoate metabolism	1/15	0.12587	0.93626
Glycosylphosphatidylinositol (GPI)-anchor biosynthesis	1/15	0.12587	0.93626
Glycerolipid metabolism	1/16	0.13372	0.93626
Fructose and mannose metabolism	1/20	0.16445	0.93626
beta-Alanine metabolism	1/21	0.17197	0.93626
Pentose phosphate pathway	1/23	0.18683	0.93626
Glycolysis / Gluconeogenesis	1/26	0.20865	0.93626
Alanine, aspartate and glutamate metabolism	1/28	0.22289	0.93626
Lysine degradation	1/30	0.2369	0.93626
Inositol phosphate metabolism	1/30	0.2369	0.93626
Porphyrin metabolism	1/31	0.24382	0.93626
Glyoxylate and dicarboxylate metabolism	1/32	0.25067	0.93626
Sphingolipid metabolism	1/32	0.25067	0.93626
Glycine, serine and threonine metabolism	1/33	0.25747	0.93626
Arachidonic acid metabolism	1/44	0.32857	1
Metabolism of xenobiotics by cytochrome P450	1/68	0.46233	1
Purine metabolism	1/70	0.47227	1

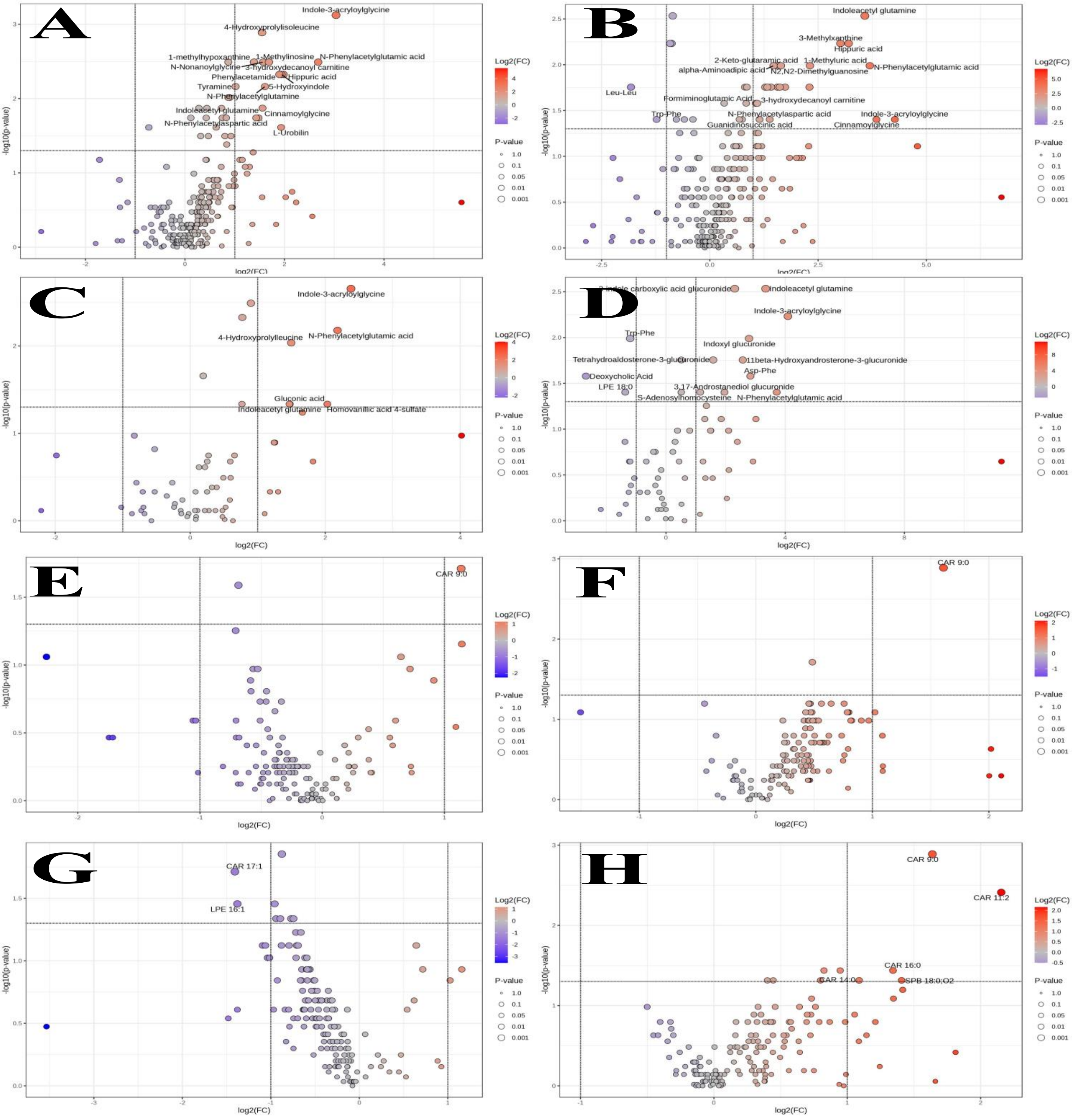

Figure S2. Volcano plots showing differences between DGF and non-DCD DGF groups: (A) metabolomic analysis in positive ionization mode on POD1, (B) metabolomic analysis in positive ionization mode on POD5, (C) metabolomic analysis in negative ionization mode on POD1, (D) metabolomic analysis in negative ionization mode on POD5, (E) lipidomics analysis - RP positive ionization mode on POD1, (F) lipidomics analysis - RP positive ionization mode on POD5, (G) lipidomics analysis - HILIC positive ionization mode on POD1, (H) lipidomics analysis - HILIC positive ionization mode on POD5.

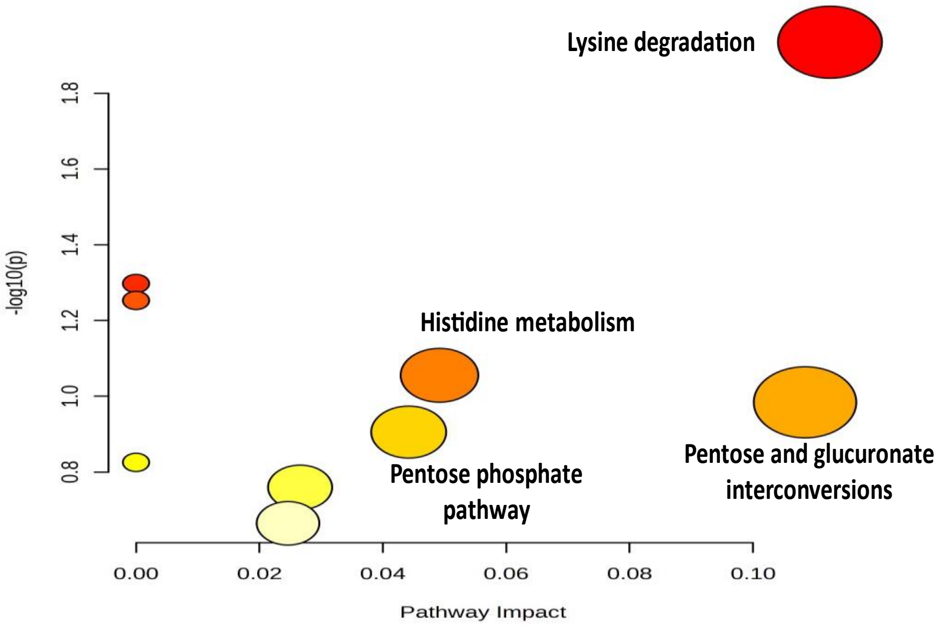

Figure S3. Pathway analysis of significantly differential features from plasma metabolomic and lipidomic analyses.

Table S4. Results of pathway analysis of significantly differential features from plasma metabolomic and lipidomic analyses.

Pathway name	Match status	p	FDR
Lysine degradation	2/30	0.011624	0.92993
Ascorbate and aldarate metabolism	1/9	0.050394	1
Caffeine metabolism	1/10	0.055851	1
Histidine metabolism	1/16	0.088015	1
Pentose and glucuronate interconversions	1/19	0.10373	1
Pentose phosphate pathway	1/23	0.12431	1
Alanine, aspartate and glutamate metabolism	1/28	0.14944	1
Cysteine and methionine metabolism	1/33	0.17392	1
Tyrosine metabolism	1/42	0.21642	1
